# A Case of Silent Mycobacterial Endocarditis Masked as Refractory Hyperparathyroidism

**DOI:** 10.7759/cureus.94657

**Published:** 2025-10-15

**Authors:** Jonathan Hurley, Gazal Arora, Samuel Ficenec, Jacob Khoury, Deena Athas

**Affiliations:** 1 Department of Internal Medicine, Tulane University School of Medicine, New Orleans, USA

**Keywords:** hypercalcemia, hyperparathyroidism, mycobacterial endocarditis, non-tuberculous mycobacteria, prosthetic heart valve

## Abstract

Non-tuberculous mycobacteria are a rare cause of infectious endocarditis and are associated with a high mortality rate. Formal recommendations guiding treatment are lacking, non-standardized, and generally rely on prior case reports. This report adds to the current literature with the intent to help guide management in similar patients. A 70-year-old female with a history of chronic, primary hyperparathyroidism and recent mitral valve replacement with an ultra-porcine bioprosthesis nine months prior presented for altered mental status secondary to hypercalcemia (total calcium: 12.5 mg/dL, free calcium: 1.84 mg/dL). Through her hospital course, it was determined she had *Mycobacteria fortuitum* endocarditis of the prosthetic mitral valve complicated by septic emboli to the brain. Antibiotics were initiated, and she was clinically stable at the time of report submission. This case highlights the need for antimicrobial susceptibility to guide management. Furthermore, many of the antibiotics utilized are known to cause serious adverse effects, decreasing their tolerability, and an important factor to consider. Complications due to non-tuberculous mycobacterial endocarditis itself can be devastating and may contribute to the high mortality rate as well.

## Introduction

Non-tuberculous mycobacterial (NTM) endocarditis is a rare pathology, with 167 cases reported since 1975 [1]. A systematic review published in 2024 found that only 21 cases of *Mycobacterium fortuitum*-specific endocarditis have been documented, with approximately 63.5% of these cases involving a prosthetic heart valve [1]. Additional risk factors for developing NTM endocarditis in native valves center on immunocompromised status and instrumentation/other procedures, including dialysis, arthroplasty, and other cardiothoracic surgeries [2-5].

Without clear guidelines on the treatment of NTM endocarditis, the majority of antibiotic regimens used include amikacin, ciprofloxacin, clarithromycin, imipenem, and/or linezolid [6]. However, these recommendations are based largely on regimens used to treat pulmonary NTM infections and corroborated mainly by individual case reports. If a prosthetic valve is believed to be the source of infection, surgical removal and replacement are indicated [1,6,7]. Rapidly growing mycobacteria, such as *Mycobacterium fortuitum* and *Mycobacterium chelonae*, are the main species causing NTM endocarditis [1]. Unfortunately, the mortality associated with these infections is high due to extended treatment durations, poor tolerability to antibiotic regimens, and the necessity of cardiac valve replacement to achieve source control [1,6]. The case fatality rate of NTM endocarditis is reported to be 44.9% [1]. *Mycobacterium* *fortuitum* survival rates vary in the literature, ranging between 22% and 25% [7,8]. This case report seeks to add to a growing body of evidence regarding therapy, given the lack of antibiotic guidelines and high mortality rates.

## Case presentation

A 70-year-old female with a past medical history of pulmonary hypertension, coronary artery disease, primary hyperparathyroidism with recurrent hypercalcemia, obstructive sleep apnea, and recent porcine bioprosthetic mitral valve replacement nine months prior, with residual mitral regurgitation, presented to the New Orleans University Medical Center from an outside hospital for hypercalcemia and altered mental status (no Mini-Mental State Examination score obtained). Additional history included progressive weakness, constipation, night sweats, reduced appetite, and a 20-pound weight loss over the previous month. Approximately two to three days before presentation, the patient had an acute worsening of confusion and disorientation. On arrival to the emergency department, the patient was febrile with a temperature of 101.5°F with other basic vital signs as follows: blood pressure, 145/82 mmHg; heart rate, 92 beats/minute; respiratory rate, 16 breaths/minute; and SpO_2_, 97% on room air. Physical examination was notable for a blowing systolic murmur loudest at the mitral post, decreased breath sounds to the left lung field, orientation to self only, and decreased strength in her extremities without other abnormal skin or nail findings. No neck stiffness or nuchal rigidity was noted.

**Figure 1:**
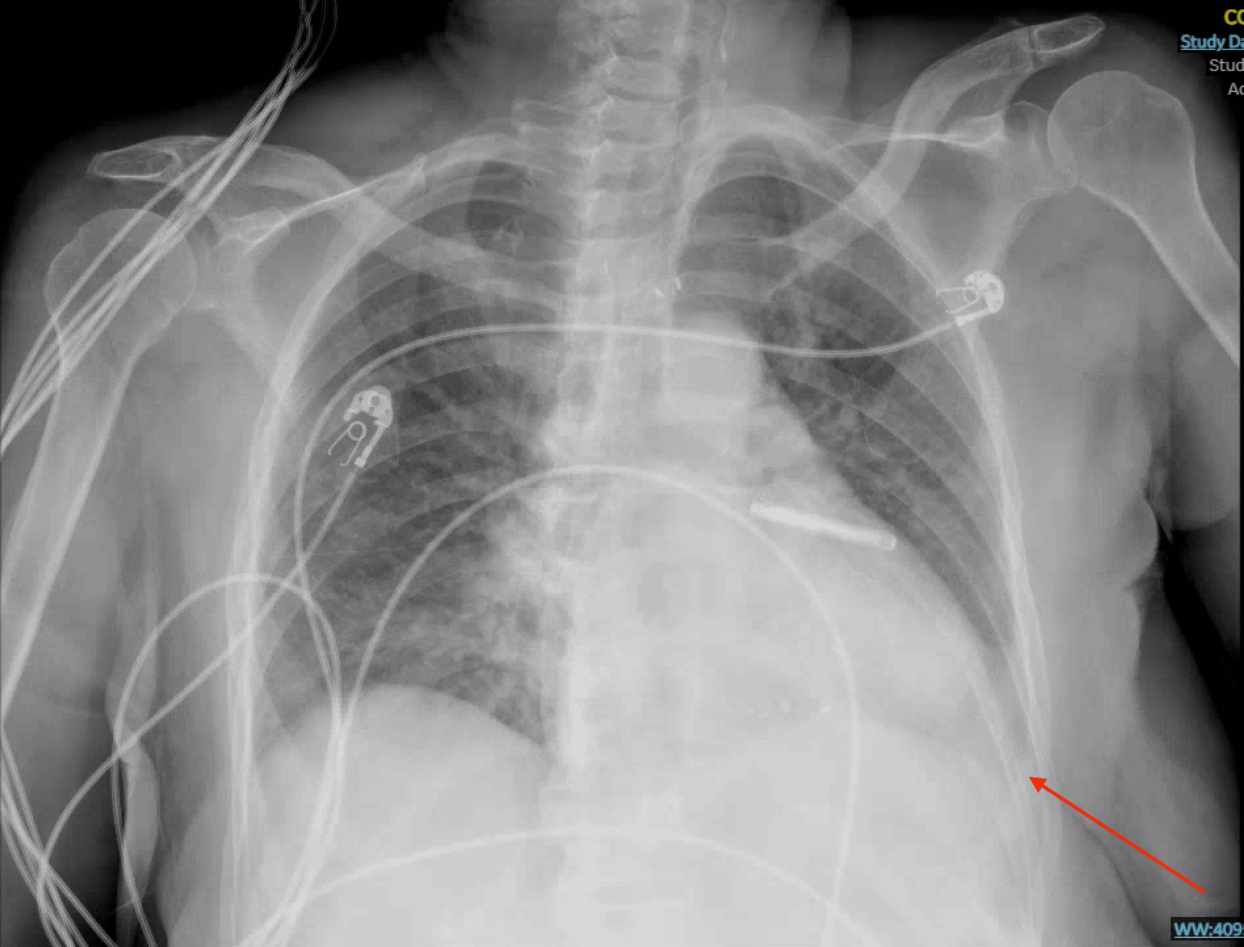
Chest X-ray on hospital day one showing left-sided pleural effusion (red arrow).

Laboratory findings were significant for a white blood cell count of 8.2 × 10^3^/µL (reference range 4.5-11 × 10^3^/µL), phosphorus of 1.4 mg/dL (reference range: 2.5-4.7 mg/dL), and brain natriuretic peptide of 743 pg/mL (reference range: <100 pg/mL). The corrected calcium was 13.4 mg/dL (reference range: 8.4-10.3 mg/dL) with an albumin of 2.9 g/dL (reference range: 3.4-5.0 g/dL) and alkaline phosphatase of 93 U/L (reference range: 20-120 U/L). Ionized calcium was 1.84 mmol/L (reference range: 1.10-1.30 mmol/L). The patient’s intact parathyroid hormone and calcitriol were elevated at 369 pg/mL (reference range: 12-65 pg/mL) and 152 pg/mL (reference range: 24.8-81.5 pg/mL), respectively, consistent with a prior history of chronic hyperparathyroidism but also concerning for a potential granulomatous process. The HIV Ab/Ag combo assay was non-reactive. No indicators of urinary tract infection were noted on urinalysis. Creatinine was normal at baseline at 1.04 mg/dL (reference range: 0.50-1.10 mg/dL). Noteworthy imaging studies included a CT of the head (Video 1) and CT angiography (Video 2) from an outside hospital without evidence of malignancy or emboli on wet read from our in-house radiologist. Additionally, the patient had a chest X-ray concerning for a left-sided pleural effusion (Figure 1). No abdominal imaging was performed.

**Video 1:** CT of the head on hospital day one without evidence of malignancy or emboli.

**Video 2:** CT angiogram on hospital day one without evidence of acute malignancy or emboli.

**Figure 2:**
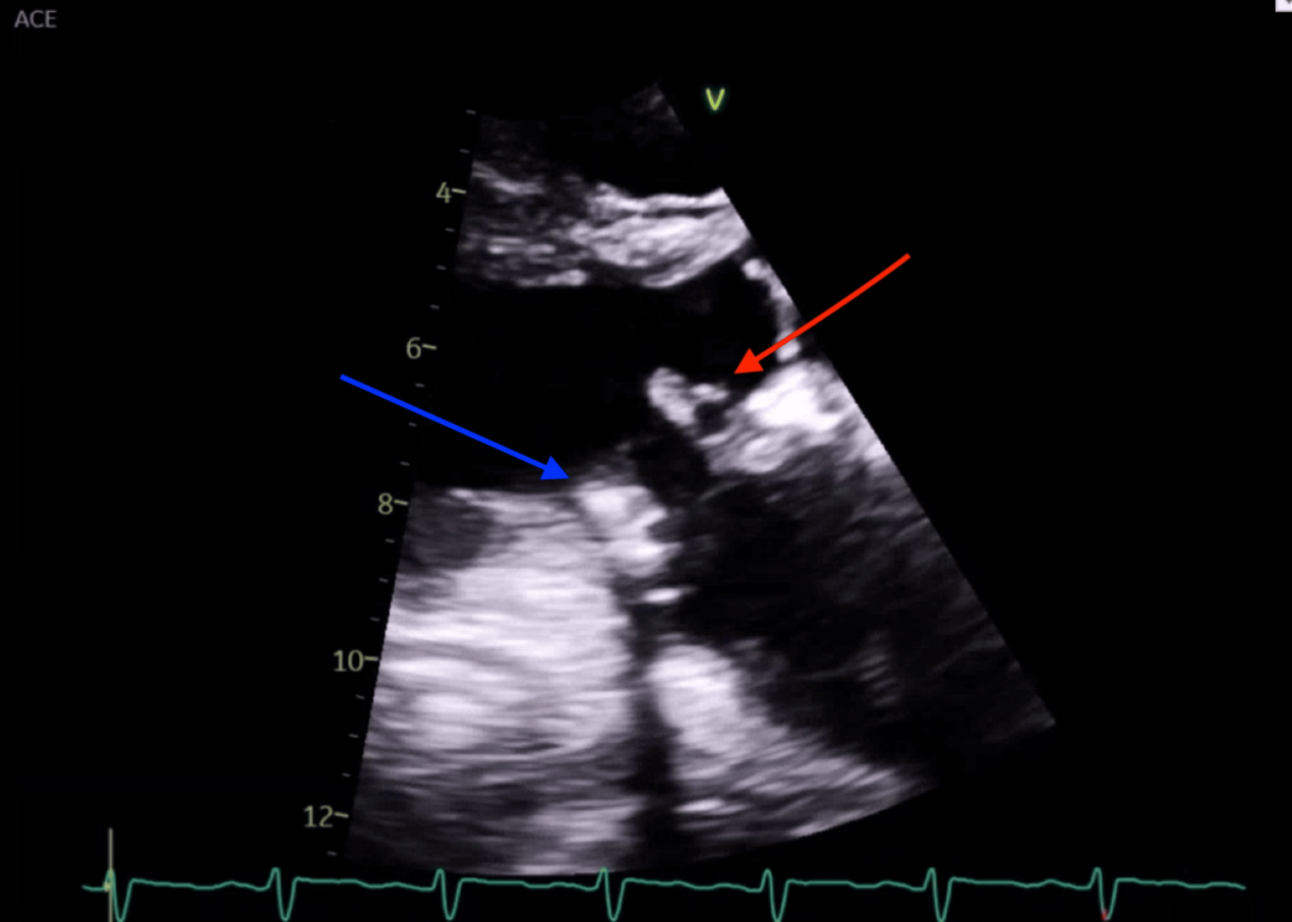
Transthoracic echocardiogram showing two highly mobile masses on hospital day 11. The mass on the anterior leaflet of the mitral valve (red arrow) with dimensions of 1.3 × 0.5 cm, concerning for infective endocarditis vs. thrombosis.

**Figure 3:**
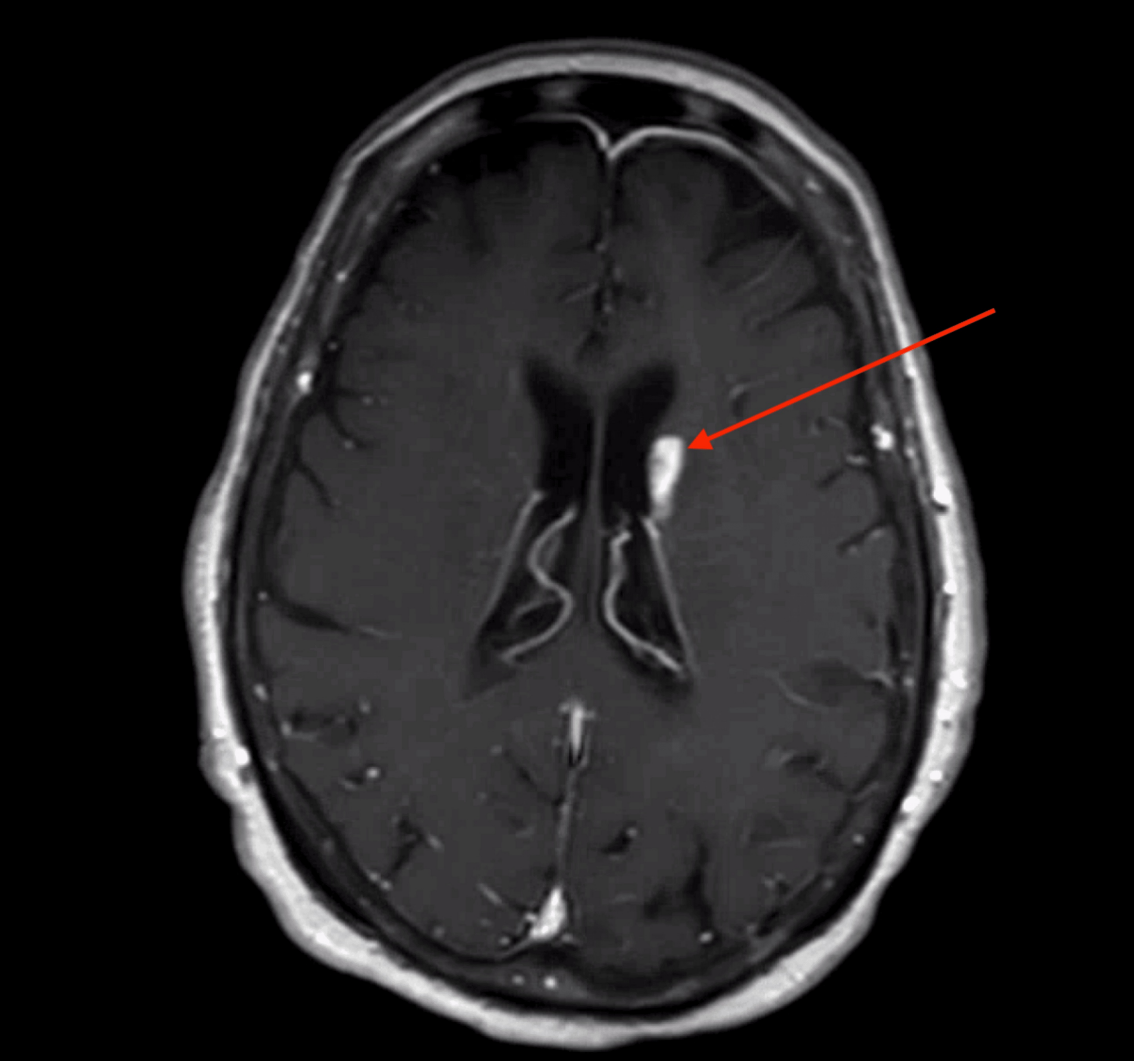
MRI of the brain with contrast (axial T1 view) showing findings concerning for septic emboli on hospital day 11. Largest lesion at the left lateral ventricle measuring 1.6 × .6 cm (red arrow).

The patient’s hypercalcemia was treated with 4 mg intravenous (IV) zoledronic acid at the outside hospital and IV calcitonin in our emergency department. She received continued treatment with cinacalcet throughout her admission, and her corrected calcium improved to within normal ranges on hospital day 12. In addition, given the finding of pleural effusion on chest X-ray and altered mental status of relatively unclear etiology, the patient was maintained on empiric antibiotics with ceftriaxone and azithromycin for potential community-acquired pneumonia. However, the patient’s mental status did not improve. By hospital day five, both sets of the patient’s initial blood cultures had resulted in acid-fast bacilli (AFB), which were speciated as *Mycobacterium fortuitum* on hospital day 11. Given this clinical picture, there was elevated concern for endocarditis. A transthoracic echocardiogram demonstrated two highly mobile masses on the prosthetic valve, which were concerning for vegetations (Figure 2). Subsequently, a transesophageal echocardiogram redemonstrated these cardiac masses and characterized them as thrombi suspicious for secondary infection. Brain MRI on hospital day 11 showed eight new brain lesions consistent with septic embolic, which were ultimately thought to be the etiology of the patient’s altered mental status (Figure 3). The patient met the 2023 Duke-International Society for Cardiovascular Infectious Diseases criteria at this time for infective endocarditis (three or more blood cultures positive for microorganisms that occasionally or rarely cause infective endocarditis, prosthetic valve present, vascular phenomena present, echocardiography showing cardiac masses on the mitral leaflets).

Infectious disease and cardiothoracic surgery were consulted to guide further management, as the mainstay of treatment for NTM endocarditis is source control and antibiotics. The patient was deemed to be a weak surgical candidate for surgical excision of her infected valve due to her significant comorbidities and concern for a poor outcome. Additionally, the patient and her family preferred not to pursue revision surgery. Because surgical source control was not possible, the infectious disease team recommended a minimum one-year duration of amikacin, imipenem-cilastin, and levofloxacin. However, these medications are known to lower the seizure threshold and cause renal injury. Given these adverse effects in combination with the patient’s baseline neurologic impairment and risk for renal insufficiency, it was thought unlikely that the patient would tolerate this therapy for the required duration. Ultimately, a goal-of-care discussion was held with the palliative care team. After discussing the risks and potential benefits, the family elected to proceed with the antibiotic regimen, understanding that full resolution of the infection was unlikely.

**Figure 4:**
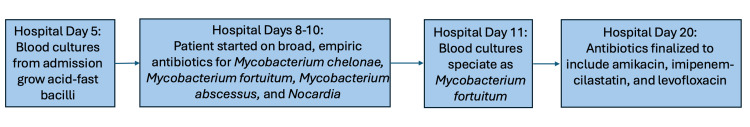
Timeline of inpatient microbiologic results and antibiotic management.

Given AFB positive blood cultures returned on hospital day five, the patient was started on empiric amikacin 1 g IV once daily on Monday, Wednesday, and Friday (MWF) (Figure 4). Her creatinine and estimated glomerular filtration rate at this time were 0.86 mg/dL (reference range: 0.5-1.10 mg/dL) and 70 mL/minute/1.73m^2^ (reference range >/=90 mL/minute/1.73m^2^). Her antibiotic regimen also included imipenem 1 g IV every 12 hours and azithromycin 500 mg by mouth every 24 hours to cover for the common rapidly growing *Mycobacterium chelonae *and *Mycobacterium fortuitum*. Cilastatin was added to imipenem 1 g IV every 12 hours on hospital day nine (Figure 4). This regimen was broadened to better cover *Mycobacterium abscessus* and *Nocardia* with linezolid 600 mg by mouth every 12 hours on hospital day 10. The blood cultures speciated *Mycobacterium fortuitum *on hospital day 11. Linezolid and azithromycin were discontinued, and levofloxacin 750 IV/PO daily was added on hospital day 14 following speciation. The patient’s antibiotic regimen was finalized on hospital day 20 to amikacin 1 g IV MWF, imipenem-cilastatin 1 g IV every 12 hours, and levofloxacin 500 IV/PO daily (Figure 4). The duration of antibiotic treatment was set for eight weeks pending follow-up. The patient was discharged on this regimen to a long-term acute care facility. At the eighth-week follow-up, susceptibilities had returned, and the patient was started on oral levofloxacin and doxycycline (Table 1). At the third-month follow-up, levofloxacin was discontinued, given QT interval prolongation to 492 ms. At about seven months post-hospitalization, the patient was clinically stable with plans for suppression with doxycycline for the remainder of her life.

**Table 1:** Antibiotic susceptibility for the Mycobacterium fortuitum group.

	Mycobacterium fortuitum group	
Antibiotics	MIC µg/mL	INTRP
Amikacin (IV)	≤8	S
Augmentin	32/16	NI
Azithromycin	>256	NI
Azithromycin (14 days)	>256	NI
Cefepime	>32	NI
Cefotaxime	>64	NI
Cefoxitin	32	I
Ceftriaxone	>64	NI
Ciprofloxacin	≤1	S
Clarithromycin	6	R
Clarithromycin (14 days)	>32	R
Clofazimine	≤0.5	NI
Clofazimine/Amikacin	≤0.5/2	NI
Doxycycline	≤1	S
Gentamicin	16	NI
Imipenem	8	I
Kanamycin	≤8	NI
Linezolid	16	I
Minocycline	≤1	NI
Moxifloxacin	≤0.5	S
Tigecycline	<=0.25	NI
Tobramycin	>16	R
Trimethoprim/Sulfamethoxazole	4/76	R

## Discussion

NTM endocarditis has increased in prevalence over the past two decades. It is most commonly seen in immunocompromised patients and those with prosthetic heart valves, and is difficult to treat with a high mortality rate [1,6,7]. Outcomes are generally poor without effective source control and in cases involving rapidly growing NTMs. *Mycobacterium fortuitum* has historically been susceptible to amikacin (90% of reported cases), imipenem (87.5%), linezolid (75%), and moxifloxacin (100%) [1]. Levofloxacin and ciprofloxacin can presumably be given interchangeably with moxifloxacin, given their degree of similarity. Different triple therapy regimens have been used in the past with varying success [1]. These include regimens such as amikacin + imipenem + moxifloxacin (57% of reported cases), amikacin + imipenem + clarithromycin (14%), amikacin + cotrimoxazole + minocycline (14%), and amikacin + cotrimoxazole + linezolid (14%).

To our knowledge, there are only two cases of survival in a patient with *Mycobacterium fortuitum* endocarditis managed non-surgically with only medical treatment. The first case was published in 2002, in which the patient was treated with oral minocycline and ciprofloxacin. They were stopped at 10 months of treatment, found to be bacteremic two weeks later, and restarted on oral doxycycline and ciprofloxacin for an indefinite duration [7]. The second case was published in 2017, and the patient was treated with two weeks of amikacin and ciprofloxacin, followed by two weeks of linezolid and moxifloxacin, and two weeks of amikacin and moxifloxacin, with subsequent moxifloxacin monotherapy for four months [8]. Thirty months after cessation of antibiotics, there were no objective findings of mycobacteremia or infective endocarditis [8]. In comparison, the patient discussed in this case was ultimately unable to tolerate therapy with fluoroquinolones (due to prolonged QT) and was treated indefinitely with doxycycline.

As seen in this patient with secondary septic embolic strokes, this phenomenon has been reported among 16.2% of patients with NTM endocarditis [1]. Other common complications of mycobacterial endocarditis include heart failure (22.8%), acute kidney injury (13.8%), and bacteremia (76%) [1]. While there is no clear evidence, one theory why these infections occur involves colonization/contamination of valves during manufacture or storage [1]. *Mycobacterium fortuitum*, in particular, has been shown to form biofilms readily, contributing to its antibiotic resistance and making it difficult to eradicate [9,10].

Of note, this case presents a complication of resistant and recurrent hypercalcemia seen in granulomatous processes consistent with mycobacterial infections. Granulomas are known to convert 25-hydroxyvitamin D to 1,25-dihydroxyvitamin D (calcitriol), causing hypercalcemia. This patient also showed signs of hyperparathyroidism (elevated intact parathyroid hormone), resistant to bisphosphonates, further clouding her diagnosis of mycobacterial endocarditis, as her altered mental status was originally explained by hypercalcemia instead of septic embolic strokes. This case highlights the importance of using parathyroid hormone and calcitriol levels to distinguish between parathyroid and non-parathyroid mediated hypercalcemia, especially when refractory to treatment.

## Conclusions

NTM endocarditis can be difficult to diagnose and difficult to treat, especially without effective source control. Empiric antibiotic regimens are currently based on treatments for disseminated and pulmonary NTM, as well as limited case reports on NTM endocarditis. Effective infection source control should be obtained early in the management course if possible. Regardless of whether the infected valve can be excised/replaced, antimicrobial susceptibility laboratory findings should guide antibiotic suppression management as soon as possible. The duration of antibiotics is extremely prolonged, and can be indefinite in many cases. Furthermore, many of the antibiotics utilized are known to cause serious adverse effects, decreasing their tolerability, and an important factor to consider when guiding management. Complications due to NTM endocarditis itself can be devastating and may contribute to the high mortality rate. Improved guidelines are needed to effectively manage these patients. Hypercalcemia is an easily forgotten manifestation of mycobacterial infections. Granulomatous processes should always be on the differential when treating a patient with subacute/chronic, bisphosphonate-resistant hypercalcemia.

## References

[1] Meena DS, Kumar D, Bohra GK, Midha N, Garg MK (2024). Nontuberculous mycobacterial infective endocarditis: a systematic review of clinical characteristics and outcomes. Open Forum Infect Dis.

[2] Kuruvila MT, Mathews P, Jesudason M, Ganesh A (1999). Mycobacterium fortuitum endocarditis and meningitis after balloon mitral valvotomy. J Assoc Physicians India.

[3] Singh M, Bofinger A, Cave G, Boyle P (1992). Mycobacterium fortuitum endocarditis in a patient with chronic renal failure on hemodialysis. Pathology.

[4] Collison SP, Trehan N (2006). Native double-valve endocarditis by Mycobacterium fortuitum following percutaneous coronary intervention. J Heart Valve Dis.

[5] Spell DW, Szurgot JG, Greer RW, Brown JW 3rd (2000). Native valve endocarditis due to Mycobacterium fortuitum biovar fortuitum: case report and review. Clin Infect Dis.

[6] Yuan SM (2015). Mycobacterial endocarditis: a comprehensive review. Rev Bras Cir Cardiovasc.

[7] Kunin M, Salamon F, Weinberger M, Genkin I, Sagie A, Tur-Kaspa R (2002). Conservative treatment of prosthetic valve endocarditis due to Mycobacterium fortuitum. Eur J Clin Microbiol Infect Dis.

[8] Gnanenthiran SR, Liu EY, Wilson M, Chung T, Gottlieb T (2017). Prosthetic valve infective endocarditis with Mycobacterium fortuitum: antibiotics alone can be curative. Heart Lung Circ.

[9] Bosio S, Leekha S, Gamb SI, Wright AJ, Terrell CL, Miller DV (2012). Mycobacterium fortuitum prosthetic valve endocarditis: a case for the pathogenetic role of biofilms. Cardiovasc Pathol.

[10] Bardouniotis E, Ceri H, Olson ME (2003). Biofilm formation and biocide susceptibility testing of Mycobacterium fortuitum and Mycobacterium marinum. Curr Microbiol.

